# Current status and future prospects of cancer-derived immunoglobulins in pancreatic cancer

**DOI:** 10.7150/jca.109497

**Published:** 2025-06-12

**Authors:** Rui Ma, Fusheng Zhang, Yongsu Ma, Weikang Liu, Guangnian Liu, Yiran Chen, Jixin Zhang, Yinmo Yang, Xiaodong Tian

**Affiliations:** 1 Department of Hepatobiliary and Pancreatic Surgery, Peking University First Hospital, Beijing, 100034, China.; 2 Department of Pathology, Peking University First Hospital, Beijing, 100034, China.

**Keywords:** cancer-derived immunoglobulin, microenvironment, pancreatic cancer

## Abstract

Immunoglobulin (Ig) is an important part of the immune system, which is mainly produced by B cells to recognize and kill pathogens. In recent years, the concept of cancer-derived immunoglobulin (cIg) has been proposed. cIg is a special form of Ig found in tumor microenvironment, and the role of cIg in tumor development and potential clinical significance of cIg have attracted more attention recently. The discovery of cIg marks a new understanding of tumor immune response and provides new ideas for early diagnosis and individualized treatment of tumors. Pancreatic cancer is a highly malignant tumor that does not respond well to conventional treatment, and causes serious complications such as pancreatic cancer-associated diabetes. Therefore, exploring potential role of cIg in pancreatic cancer and the progression of pancreatic cancer-associated diabetes is expected to be a breakthrough to improve the diagnosis and treatment of pancreatic cancer and associated complications. This review aims to summarize current research status of cIg in the field of pancreatic cancer, and provide new ideas for modulating microenvironment of pancreatic cancer to improve diagnostic efficiency and therapeutic effect.

## Introduction

In classical concept of immunology, Ig is produced only by B cells, which comprises two immunoglobulin heavy (IgH) chains and two immunoglobulin light (IgL) chains 1. IgH is divided into Igμ, Igγ, Igα, Igδ, Igε, Igγ five types and IgL is divided into Igκ and Igλ(1). The C-terminal regions of the IgH and IgL chains are constant in their sequences, which called constant (C) regions. The N-terminal regions are called variable (V) regions, which represent the recognition ability of multiple antigens through molecular mechanisms including V(D)J recombination, somatic hypermutation (SHM) and class-switch recombination (CSR) 2. According to the classification of IgH, Ig is classified into five types: IgM, IgG, IgA, IgD, and IgE. B-cell derived Ig is an important part of humoral immunity, and can mediate antibody-dependent cellular cytotoxicity (ADCC), complement-dependent cytotoxicity (CDC) and antibody-dependent cellular phagocytosis (ADCP) by neutralizing antigens 3. Different classes of B-cell derived Igs affect the progression of tumors, and four human IgG subclasses have been identified 4. IgG1 has an important killing effect on tumor cells, and its structural instability is considered to be an important factor leading to immune escape in tumor microenvironment (TME) 5. The Fc domain of IgG1 can bind to the FcR of natural killer (NK) cells or complement, mediating ADCC, ADCP and/or CDC, which triggers the cleavage of tumor cells 6.

In recent years, proteases in tumor immune microenvironment (TIME) have been identified that can disable the function of IgG immune effect. Selective cutting of hinge area leads to local damage of IgG, which cannot interact with NK cells or complement, and promotes the survival and proliferation of tumor cells 6. In breast cancer tissues the cleavage of IgG1 by tumor-related hydrolase can block the recruitment of macrophages and immune escape of tumor cells 5. However, IgG4 plays a role in promoting tumor progression. Studies have shown that elevated levels of IgG4 in tissues and serum are associated with poor prognosis of cholangiocarcinoma, gastric cancer, colorectal cancer and malignant melanoma 7,8. IgG4 binds to the inhibitory FcγRⅡb receptor, which inhibits both innate immunity (activation of macrophages, mast cells, and basophils) and adaptive immunity (activation of DC and cross-antigen presentation) 9,10. Moreover, IgG4 promotes the transformation of macrophages to the M2b-like state, which can secrete chemokines (C-C motif), ligand 1 (CCL1), and interleukin-10 (IL-10) to support regulatory cell recruitment and further shape a tolerogenic microenvironment 9. IgE is a key mediator of type I hypersensitivity and anti-parasitic immune response, and exert anti-tumor effects by inducing macrophage polarization 11. When macrophages cross-link with IgE, macrophages reprogram from M0 or M2 to M1, and finally achieve anti-tumor effect 12. It has been found that Fc fragment of IgM receptor (FcmR) expressed by mononuclear macrophages in myeloma promotes tumor growth. The activation of FcmR inhibits antigen uptake and maturation of dendritic cells, affects antigen presentation, inhibits T cell activation. Therefore, blocking FcmR through targeted therapy can inhibit tumor growth and invasion 13.

The presence of an "Ig-like" protein was first identified using anti-human IgG antibodies and protein A in several types of cancer cells 14. Later studies confirmed that IgG and other Ig isotypes were found in various cancer cells, and collectively named cancer-derived Ig (cIg) which is associated with poor prognosis of multiple types of tumors 15-20. CIg is mainly involved in three aspects of tumorigenesis and progression, including: promoting tumor cell growth and proliferation, promoting tumor cell migration and invasion and aiding tumor immune escape 21,22. In addition, other functions of cIg include affecting TME, promoting tumor cell drug resistance, promoting tumor-associated chronic diseases, affecting tumor-associated thrombosis, which lead to poor prognosis of tumor patients 23-25.

Pancreatic cancer is one of the malignant tumors with the worst prognosis 26. In particular, immunotherapy of pancreatic cancer is challenging, mainly due to immunosuppressive TME of PDAC characterized by poor infiltration of effector T cells, prominent myeloid inflammation, and a low mutational burden predicted to generate very few immunogenic antigens 27. A deep understanding of cIg in pancreatic cancer may become a breakthrough to improve the effect of immunotherapy for PDAC. Therefore, this review aims to summarize current status of cIg research in the field of PDAC, and provide new ideas for improving the effect of immunotherapy of PDAC.

## Structural characteristics of cIg

cIgs are abundant in tumor patients, including almost all types of IgH. However, the composition of cIgs is not exactly the same as that of B-cell derived Igs 28. At the subcellular level, cIg is primarily located in the cytoplasm and cell membrane, and it can be detected in secreted form in the supernatant of cancer cells. RAG1 and RAG2, as essential proteins for V (D) J rearrangement, have been detected in a variety of tumor cell lines, confirming the ability of cancer cells for V(D)J rearrangement. Zheng *et al.* found that Variable-Diversity-Joining gene segment of heavy chain (VHDJH) transcripts from cIg was similar to those from B lymphocytes, but showed different characteristics 29.

**Figure 1:**
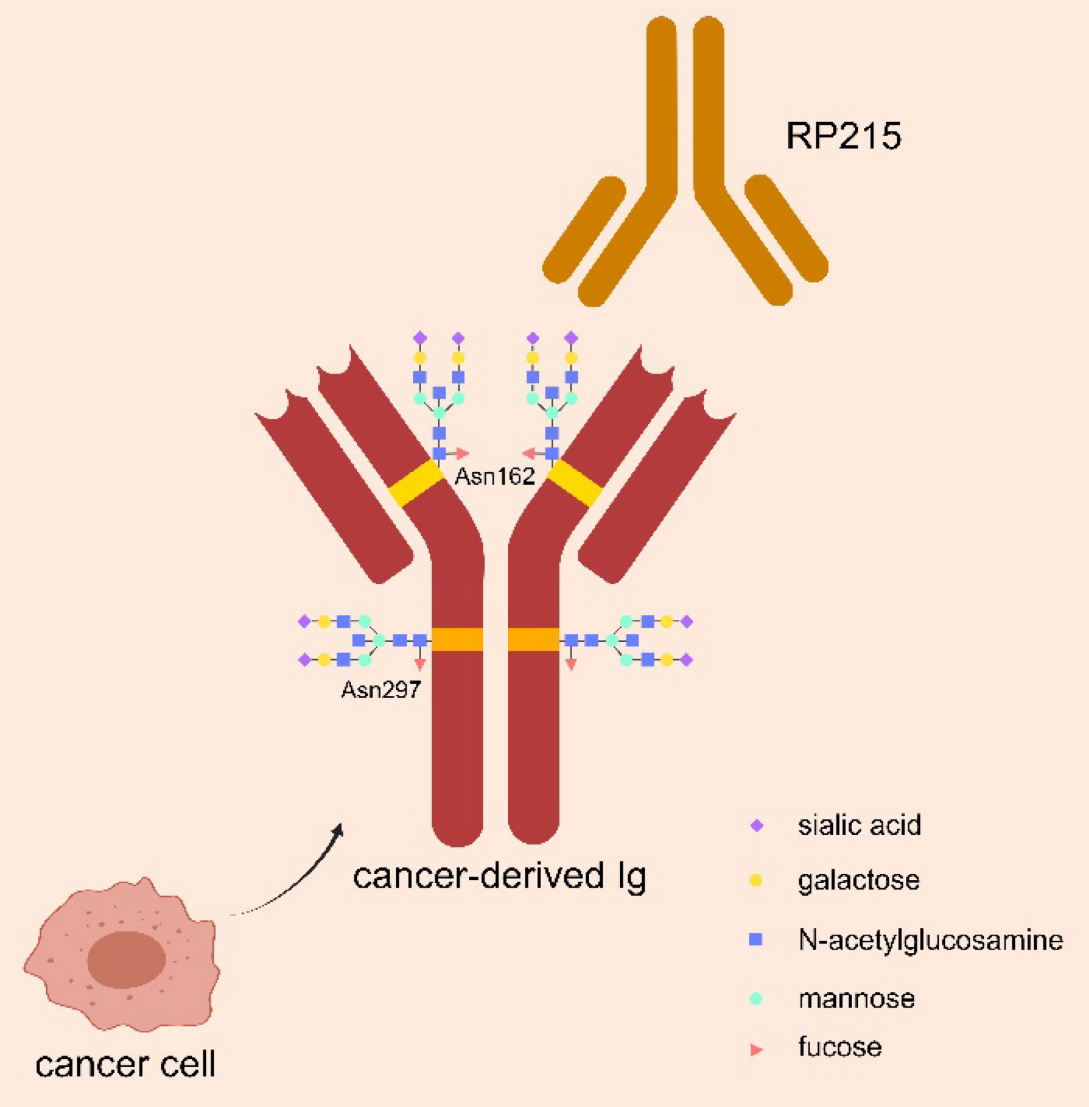
** Special glycosylation structure of cIg.** CIg has a special sialylated N-glycan structure at Asn162 site, which can be specifically recognized by the monoclonal antibody RP215 to distinguish it from B-cell-derived Ig. The classical salivated N-glycan structure of Asn297 site in Ig Fc fragment is also demonstrated, which is responsible for maintaining Ig function.

Glycosylation is an important modification to regulate biological function of Ig, by playing role in intercellular communication, cell-matrix interactions, and immune regulation. As shown in Figure 1, N-glycosylation of asparagine 297 (Asn297) is a consensus glycosylation event responsible for maintaining IgG function, which is located in the CH1 domain of IgG heavy chain and carries sialic acid modifications 30, 31. RP215 is a monoclonal antibody that can identify Asn162, a specific glycosylation site of cIg, but could not bind to B-cell derived Ig. RP215 can be used to distinguish cIg from B-cell derived Ig 32.

## CIg promotes malignant behaviors in cancer cells

CIg promotes the malignant behaviors, such as growth, proliferation and invasion of cancer cells through various mechanisms, and is associated with poor prognosis of tumor patients. The expression of Igκ mRNA was significantly increased in atypical hyperplasia and carcinoma tissues compared to epithelial cells with cervicitis, which may be a potential marker for malignant transformation of cells 33. The use of antisense DNA or anti-human immunoglobulin antibodies to block cIg can inhibit the growth and survival of cancer cells. Meanwhile, anti-human IgG antibodies to inhibit cIgG in immunodeficient nude mice showed inhibitory effects on cancer cells, supporting the role of cIg in promoting cancer growth. In addition, cancer cells with high IgG expression were found to exhibit cancer stem cell-like properties, such as co-expression of CD44v6, high sphere-forming capability, and resistance to chemotherapy 34.

The potential cIg interacting proteins in cancer cells were detected by co-immunoprecipitation, and RACK1, RAN and PRDX69, which are related to cell growth and oxidative stress, were confirmed to interact with cIg 35. In addition, Igκ and Igλ can maintain high expression of the anti-apoptotic molecule Bcl-xL, enhancing the anti-apoptotic ability of cancer cells 36. Zheng *et al.* demonstrated in cervical cancer and nasopharyngeal carcinoma that epithelial cancer cells expressed Igα heavy chains, and cIgA increased the percentage of synchronous tumor cells that enter the S phase from early mitosis 37. These results reveal a new mechanism of cancer cell proliferation and provide a new way to inhibit malignant transformation of cells.

## CIg inhibits anti-tumor function of immune system

cIgG promotes immune escape of tumor cells through T lymphocytes in TME 38. In mouse melanoma models, cIgG with sialylation can inhibit T cell proliferation by reducing the frequency of CD4+ and CD8+T cells in tumor tissues, thereby promoting tumor growth. It is noteworthy that a variety of inhibitory sialic acid-binding immunoglobulin-like lectin (Siglec) is highly expressed on CD4+ and CD8+ T cells, transmitting inhibitory signals to immune cells 38. In a study using breast cancer, ovarian cancer, and lung squamous cell carcinoma *et al.*, cIg was found to be an important ligand of Siglec, and its inhibitory effect on effector T cells depends on the sialylation 38. Siglecs-15 is highly expressed in effector T cells of cancer patients and promotes metastasis of cancer cells, but is less expressed in effector T cells of healthy people, suggesting its potential as an effective anti-tumor target 38.

ADCC is an important anti-tumor immune mechanism by which effector cells actively kill target cells bound by specific antibodies. Hu *et al.* analyzed ADCC activity of cIg and found that cIg can bind to the Fc receptor (FcR) of monocytes and natural killer (NK) cells through Fc domain, and then show weaker ADCC with effector cells 33. They speculate that cIg can compete with B-cell-derived Ig for effector cell FcR, thereby inhibiting ADCC and facilitating tumor immune escape 33. In addition, Li *et al.* found in cervical cancer that cIg can reduce ADCC effect induced by antibodies against human epithelial growth factor receptor (EGFR) 39.

## CIg plays a pro-tumor role through a variety of other mechanisms

Tumor-associated thrombosis is a major risk factor for death in cancer patients, who often have abnormal platelet activity. cIgG could activate platelet FcγRⅡa signal to activate platelets, thus playing an important role in malignant progression of tumors 40. In addition, cIg may increase tumor susceptibility. Tx is a human nasopharyngeal carcinoma transforming gene extracted from the CNE2 genomic DNA library of nasopharyngeal carcinoma (NPC) cell line. Sequence analysis showed that Tx encodes an abnormal Igκ light chain 41. Two single nucleotide polymorphism (SNP) loci, rs232230 (5658C/G), and rs232228 (3635T/C)(66) were positively correlated with the susceptibility to nasopharyngeal, gastric and breast cancer 41. Notably, cIgG expression was significantly upregulated when cervical cancer cells were exposed to lipopolysaccharide (LPS), suggesting that cIgG may be involved in the regulation of Toll-like receptor (TLR) 4 signaling, thereby promoting cervical cancer cell proliferation 35. These results suggest that cIg may be a novel therapeutic target for the treatment of inflammation-mediated cancers 42,43. In addition, free light chain (FLC) of Ig plays a catalytic role in colitis-associated colon cancer by activating inflammasome, associated with increased levels of cleaved caspase-1, IL-1β, and IL-18 44.

## The role of cIg in pancreatic cancer

In 2011, Li *et al.* described IgG expression in pancreatic cancer based on proteomic analysis of pancreatic cancer tissue and normal tissue 45. In 2015, Wan *et al.* used immunohistochemistry and in situ hybridization to detect the expression of IgG heavy and light chains in 50 cases of human pancreatic cancer, and further verified the expression of Ig in pancreatic cancer at the tissue level. Notably, they found that Ig signals were also detected in adjacent islet tissue. In addition, they conducted extensive validation at the cellular level, detecting the presence of Ig in multiple pancreatic cancer cell lines 46.

### CIg as a diagnostic marker in PDAC

Unlike normal islet tissue-derived Ig, pancreatic cancer derived IgG showed unique sialylation at asparagine 162 (Asn162) in the Fab region, and sialylated-IgG (SIA-IgG) is the main functional component of cIg 38. Since cIgs have specific glycation epitopes that can be recognized by RP215, RP215 is an immune probe to study the expression of cIg as a serum pan-cancer marker that can improve tumor diagnosis.

In addition to the detection of cIg in the serum of pancreatic cancer patients, it is demonstrated that cIg and its mRNA can be detected locally in pancreatic cancer through immunohistochemistry (IHC), Western blotting, polymerase chain reaction (PCR), and in situ hybridization 46. The absence of IgG-specific receptors including CD16, CD32, CD64, and FcRn in pancreatic cancer cells suggests that IgG is produced by these cells rather than being absorbed from surrounding tissues or circulation 46. The detection of IgG mRNA in pancreatic cancer cells further supports the concept that Ig is locally synthesized by tumor cells 45.

### Prognostic value of cIg in PDAC

Kaplan-Meier analysis showed that disease-free survival (DFS) and overall survival (OS) were significantly shorter in patients with high cIgG expression than with low expression. Multivariate Cox regression analysis identified high cIgG expression as an independent prognostic factor for DFS and OS. In vitro studies demonstrated that cIgG knockdown suppressed the proliferation, migration and invasion of PDAC cells 19. These results demonstrate the significance of cIg in the prognosis of pancreatic cancer.

To increase the possibility of radical resection and achieve systemic tumor control at an early stage, more patients with PDAC receive neoadjuvant therapy (NAT). Patients with negative expression of cIgG in fine needle aspirate (FNA) samples had longer survival and were good independent predictors of poor pathological response (PR), with sensitivity and specificity of 63.9% and 80.6%, respectively 17. The expression of cIg in FNA samples is a new potential biomarker of NAT response in PDAC patients, which is expected to be used to identify patients who benefit most from NAT 17.

### CIg promotes the proliferation and invasion of pancreatic cancer cells

Recent studies have identified potential targets for pancreatic cancer therapy due to their role in the regulation of pancreatic cancer cell proliferation and invasion 47-49. Interestingly, the most important functional subtype of Ig expressed by pancreatic cancer cells, Igγ-1 chain C region (IGHG1), may be involved in tumor cell proliferation and immune escape mechanism 45. In addition, the proliferation, migration and invasion ability of human pancreatic cancer cell lines BxPC-3 and T3M4 were significantly inhibited by inhibiting cIgG 19.

### CIg promotes immune escape of pancreatic cancer

In vitro cytotoxicity assay showed that IGHG1 can competitively bind to Fcγ receptors (FcγR) on the surface of NK cells, and assist immune escape of tumor cells by blocking the activity of NK cells to downregulate the ADCC effect 45. In addition, it has been demonstrated that PDAC cIgG may be involved in the polarization and function of tumor-associated macrophages (TAMs) in TME 50. In the presence of cancer cell fragments, cIgG-induced activation of FcγRⅠ/Ⅲ signaling enhanced NF-κB signaling and promoted IL-1β production. In animal models, IL-1β has been shown to increase peritoneal metastasis and distant metastasis to the lung and liver 50. This may be a potential mechanism by which cIg is associated with poor prognosis. Based on the role of cIg in TME, it is expected to explore immunotherapy strategies targeting cIg to improve the efficacy for pancreatic cancer treatment.

### cIg is involved in the progression of pancreatic cancer-associated diabetes

cIg is related to the pathogenesis of pancreatic cancer-associated diabetes 46. New diabetes in patients with pancreatic cancer is characterized by B-cell dysfunction and severe peripheral insulin resistance, suggesting that its pathogenesis is complex and may not be caused simply by destruction of islet cells or obstruction of pancreatic ducts by tumor cells 51. The amount of IgG expression in islet cells adjacent to tumor cells correlated with the distance from tumor cells, suggesting that tumor cells may also induce IgG expression in neighboring endocrine cells 46. When islet cells are induced to synthesize IgG, the production of insulin and other hormones may be negatively affected, which may be an explanation for reduced insulin secretion in patients with pancreatic cancer and tumor-related diabetes, but further studies are needed to evaluate the mechanism of cIg function and regulation.

## Conclusions and Perspectives

**Figure 2:**
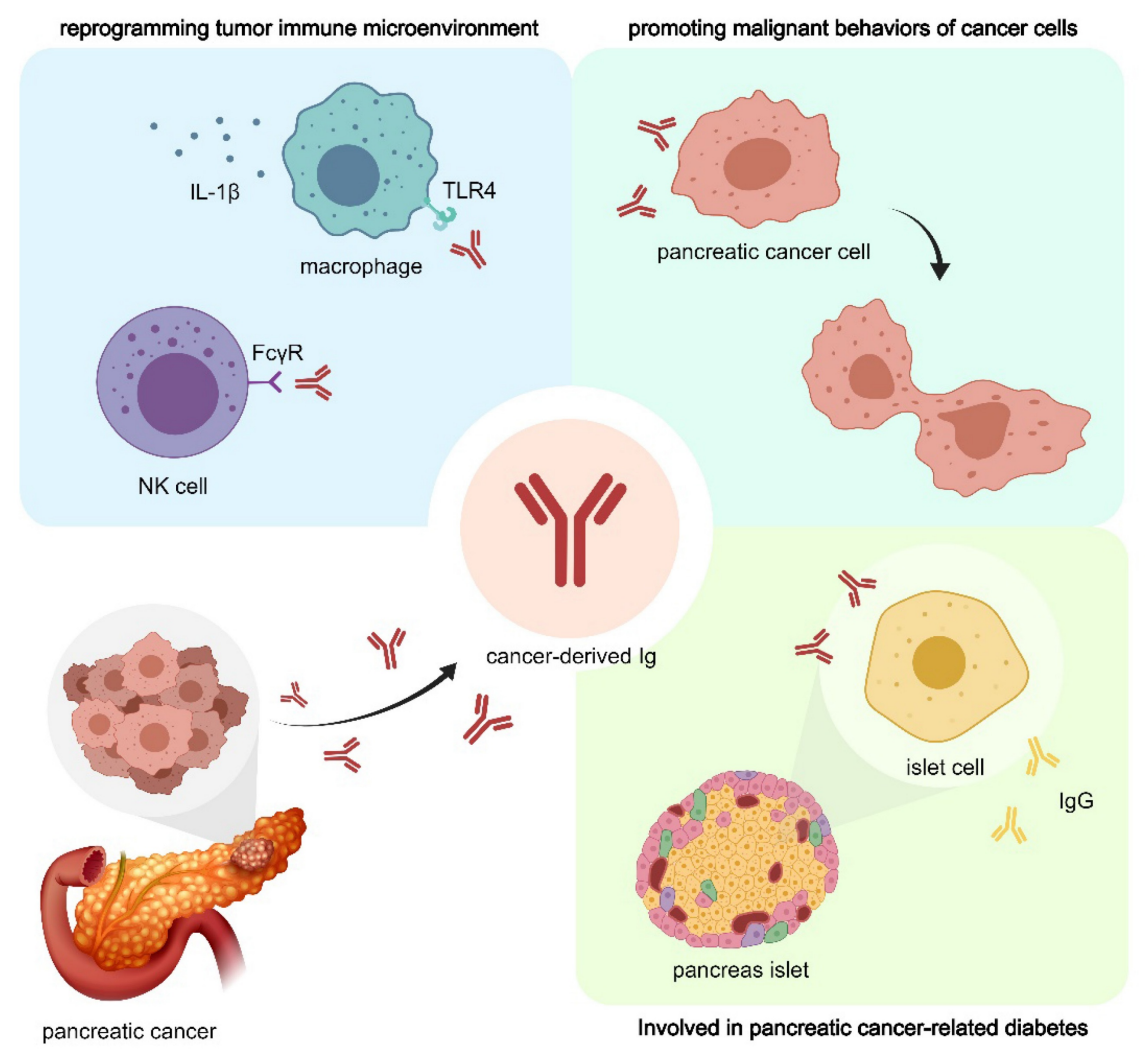
** cIg plays important role in pancreatic cancer through multiple mechanisms.** CIg can directly promote the proliferation and invasion of pancreatic cancer cells. In addition, CIg can interact with NK cells and macrophages, participate in the reprogramming of PDAC immune microenvironment, and promote tumor immune escape. cIg promotes IgG expression in adjacent islet cells and affects normal hormone secretion, thus participating in the progression of pancreatic cancer-associated diabetes.

To sum up, the role of cIg in TME has been paid much attention in the field of tumor therapy. cIg is found to be highly expressed in pancreatic cancer cells and could be a powerful prognostic marker for patients with pancreatic cancer and can predict the efficacy of NAT to a certain extent 17. As shown in Figure 2, cIg promotes pancreatic cancer cell proliferation and invasion, assist tumor cell immune escape, and promote pancreatic cancer-related chronic diseases 22. These findings suggest the development of new targets aimed at selectively blocking cIg for future diagnosis and treatment of pancreatic cancer.

Although current research on cIg in the field of pancreatic cancer shows promising prospects, the underlying molecular mechanism is still unclear due to the complexity of pancreatic cancer tumor types and microenvironment 52, 53. The specific mechanisms and downstream targets of Ig secretion regulation in pancreatic cancer cells remain to be further explored. The role of cIg in the regulation of cell adhesion of pancreatic cancer cells remain unclear 54. In addition, the crosstalk between cancer-derived sialylated IgG (SIA-IgG) and Siglecs receptors that recognize sialic acid in tumor immune evasion and pancreatic cancer immunotherapy need further investigations 55, 56. Interestingly, a recent study reported that sialylated IgG-integrin β4-FAK-Src-Erk-p300-c-Myc pathway could promote liver metastasis of colorectal cancer 57. Considering that the liver is the most common site for pancreatic cancer metastasis, it is urgent to examine whether similar pathway mediates the role of cIg in pancreatic cancer metastasis. Finally, it is important to explore clinical application of cIg in the diagnosis and treatment of pancreatic cancer. For example, the expression of SIA-IgG has been shown to be associated with poor differentiation, metastasis, and chemoresistance in pancreatic cancer 15. CIg may be a breakthrough in addressing the dilemma of unique inhibitory immune microenvironment of pancreatic cancer. At present, preclinical studies on cIg have shown promising prospects, exploring potential molecular targets to block the tumor promoting effect of cIg, or combining with currently known systemic therapies, will provide new ideas for effective therapy of pancreatic cancer.

### Funding

This study was supported by National Natural Science Foundation of China (No. 82271764, 82171722 and 82471772), the National Key Research and Development Program of China (2021YFA0909900, 2023YFC2413400), Beijing Natural Science Foundation (L246015), National High Level Hospital Clinical Research Funding (Interdepartmental Research Project of Peking University First Hospital 2023IR23, 2024IR11), National High Level Hospital Clinical Research Funding (Scientific Research Seed Fund of Peking University First Hospital 2023SF47), National High Level Hospital Clinical Research Funding (Youth Clinical Research Project of Peking University First Hospital 2023YC06), and Research and Translational Application of Clinical Characteristic Diagnosis and Treatment Techniques in the Capital (Z221100007422070).
